# Knowledge and risk factors for foot-and-mouth disease among small-scale dairy farmers in an endemic setting

**DOI:** 10.1186/s13567-019-0652-0

**Published:** 2019-05-14

**Authors:** Dickson Machira Nyaguthii, Bryony Armson, Philip Mwanzia Kitala, Beatriz Sanz-Bernardo, Antonello Di Nardo, Nicholas Anthony Lyons

**Affiliations:** 1 Department of Public Health, Pharmacology and Toxicology, Faculty of Veterinary Medicine, University of Nairobi, P.O. Box 29053, 00625 Kangemi, Kenya; 2 The Pirbright Institute, Ash Road, Pirbright, Woking, GU24 0NF UK; 3 Institute of Biodiversity, Animal Health and Comparative Medicine, College of Medical, Veterinary and Life Sciences, University of Glasgow, Graham Kerr Building, Glasgow, G12 8QQ UK; 4 European Commission for the Control of Foot-and-Mouth Disease (EuFMD), Food and Agriculture Organization of the United Nations, Viale delle Terme di Caracalla, Rome, Italy; 5 Department of Veterinary Public Health, Pharmacology and Toxicology, Faculty of Veterinary Medicine and Surgery, Egerton University, P.O. Box 536-20115, Egerton, Kenya

## Abstract

Foot-and-mouth disease (FMD) is a highly contagious viral infection of cloven-hoofed animals. In Kenya, the disease is endemic with outbreaks typically occurring throughout the year. A cross-sectional study was undertaken in Nakuru County to investigate farmer knowledge and risk factors for clinical disease. Semi-structured interviews were conducted on 220 smallholder farmers, selected using random spatial sampling. The majority of respondents (207/220 [94.1%]) knew of FMD and 166/207 (80.2%) of them could correctly identify the disease based on their knowledge of the clinical signs. Forty-five out of 220 farmers (20.4%) vaccinated their livestock against FMD in the previous 6 months, although of those who knew of FMD only 96/207 (46.4%) perceived it as a preventive measure undertaken to reduce the risk of disease in their farm. FMD had occurred in 5.9% of the surveyed farms within the previous 6 months (from May to November 2016). Using multivariate analysis, the use of a shared bull (OR = 9.7; *p* = 0.014) and the number of sheep owned (for each additional sheep owned OR = 1.1; *p* = 0.066) were associated with an increased likelihood of a farm experiencing a case of FMD in the previous 6 months, although the evidence for the latter was weak. This study reports risk factors associated with clinical FMD at the farm level in a densely populated smallholder farming area of Kenya. These results can be used to inform the development of risk-based strategic plans for FMD control and as a baseline for evaluating interventions and control strategies.

**Electronic supplementary material:**

The online version of this article (10.1186/s13567-019-0652-0) contains supplementary material, which is available to authorized users.

## Introduction

Foot-and-mouth disease (FMD) is a viral disease affecting cloven-hoofed animals. The causative pathogen, FMD virus (FMDV), belongs to the family *Picornaviridae* and genus *Aphthovirus* [1]. The disease causes major economic losses in dairy production [2]. In Kenya where the disease is endemic [3], FMD was ranked second among infectious diseases of livestock with the highest impact on pastoralist livelihoods [4].

Kenya has the largest developed smallholder dairy farming system in sub-Saharan Africa [5] and the sector contributes 70% of all milk produced in the country [6]. Nakuru County is located in the central highlands of Kenya where dairy farming is an important economic activity [7]. Clinical FMD in this area has been regularly reported through field investigations conducted during “real-time” training courses conducted every year by the European Commission for the Control of Foot-and-Mouth Disease (EuFMD).

Putative risk factors for clinical FMD among cattle have been investigated in a variety of endemic settings. Commonly reported risk factors include: the communal sharing of water or feed [8], the type of livestock production system, the number of calves aged up to 6 months present in the holding, and the presence of small ruminants [9–12]. Additional risk factors identified include: the distance of the farm to major roads [12], the frequency of cattle purchased [8], animals residing in an area with history of FMD in the last 12 months [13], and animals owned by livestock traders [13].

An understanding of these risk factors at country level is an important component of developing a national risk-based control strategy required to progress towards stage 2 of the Progressive Control Pathway for FMD control [14]. Kenya is currently in stage 1, which involves collecting information “to gain an understanding of the epidemiology of FMD in the country and develop a risk-based approach to reduce the impact of FMD” [14]. A control strategy has been developed but has not been fully implemented and is undergoing revision in line with the devolution of veterinary authority to the County level.

FMD vaccination in Kenya is not compulsory; private farmers are entitled to have their animals vaccinated either by hiring private animal health practitioners or through subsidised government vaccination exercises, if sufficient vaccine is available. Although the County government in Nakuru utilises vaccination for FMD control, the scope is limited to a reactive “ring-based” strategy in response to confirmed outbreaks. Despite this, large-scale farms may perform routine vaccination [15]. The currently available vaccines are either monovalent or polyvalent containing a combination of strains from O, A, SAT 1 and SAT 2 serotypes and with at least a 6.0 PD_50_ (50% protective dose). A recent study has found these serotypes to be the most prevalent in Nakuru County for the period from 2010 to 2016 [16].

The Kenyan government’s “Vision 2030” recognises that livestock play a very important role in the national economy [17]. In this context, control of infectious diseases of livestock (including FMD) is seen as a pathway to accelerating productivity in the sector with the potential to alleviate poverty [18]. Despite the importance of smallholder dairy farmers to the national milk output and the potential high impact of FMD on productivity, no study has focused on quantifying risk factors for clinical disease in this sector. Knowledge and practices of smallholder dairy farmers in relation to FMD is also poorly quantified. This study aimed to contribute to this knowledge gap by analysing data collected from a cross-sectional survey among smallholder dairy farms within Nakuru County, Kenya.

## Materials and methods

### Study area

Nakuru County is found in the mid-west area of Kenya with an elevation of approximately 1850 m above sea level, and characterised by an average rainfall of 963 mm per year. The area is home to a national park (Lake Nakuru National Park) and a forest reserve (Mau forest reserve) hosting wildlife. National statistics from the 2009 Kenya Housing and Population Census reported Nakuru County as having a total of 409 836 households [19], 439 994 cattle, 502 035 sheep, 227 037 goats, and 13 894 pigs [20].

**Figure 1:**
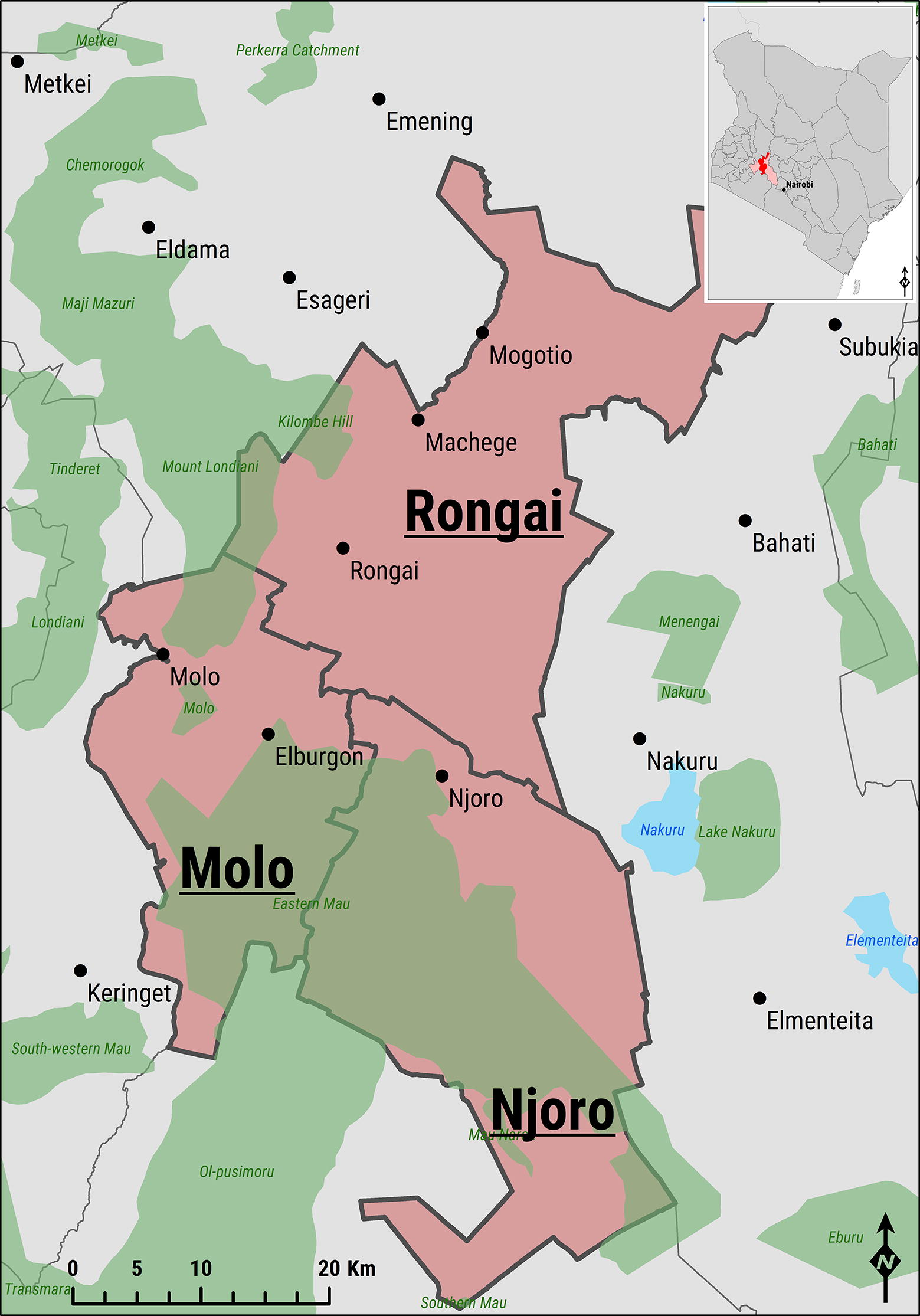
**Map of the study area.** Salmon-coloured admin regions indicate the location of the Nakuru County within Kenya and each of the sub-counties of Molo, Njoro and Rongai targeted for the study. Green-shaded areas represent protected areas, whilst those in azure define lakes.

This study was performed as part of a larger project investigating the use of milk from pooling facilities for FMDV surveillance. The study area consisted of the catchment areas of five neighbouring milk-pooling facilities located within Molo, Njoro and Rongai sub-counties of Nakuru County, Kenya (Figure 1). All catchment areas either bordered each other or overlapped so a single spatial polygon was created using Google Earth (Google Inc., USA) and exported to QGIS version 2.18.10 (QGIS Development Team, Las Palmas, USA).

### Study design

A cross sectional study design was used whereby data regarding farmers’ knowledge, occurrence of clinical FMD and putative risk factors were collected and analysed. This represents a cost-effective methodology for generating hypotheses that could be subsequently used as part of larger studies in the area.

The study population was small-scale dairy farmers in Nakuru County, Kenya located within the catchment area of the milk-pooling facilities. Inclusion criteria were: (i) premises with at least one but no more than fifty cattle at the time of the interview, and (ii) having the cattle located in the proximity of the household (i.e. not farmed at another premises). Farms were selected by spatial sampling using QGIS to generate random points within the defined study area. The list of geo-coordinates was uploaded onto GPS units (Garmin eTrex 10, Garmin Corp., UK) which were used to locate the points on the ground. The nearest smallholder farm to the generated random point was selected for the interview. If it did not meet the inclusion criteria, the next closest premises was approached. Coordinates indicating areas where no obvious closest smallholder farm could be identified (e.g. in the centre of a large-scale farm or woodland/plantation) were removed from the study.

### Sample size calculation

The sample size was based on the estimated number of farms affected by FMD in the previous 6 months and calculated using the formula for sampling binomial outcomes [21]

$$n = Z_{\alpha }^{2} \times \frac{{P\left( {1 - P} \right)}}{{L^{2} }}$$

where *n* is the required sample size, *P* is the expected proportion of households being affected in the previous 6 months and *L* is the desired precision at a *Z* confidence level (corresponding to α = 95%). The estimated prevalence was set as 15% (based on experience of one of the authors [NL] doing surveys in the area as part of EuFMD training courses) with an absolute precision of 2.5%. This estimated prevalence was used because there was nothing to refer to in the literature or from any other records on the prevalence of clinical disease in the area. This resulted in 197 farms to be interviewed. The sample size was inflated by 20% to account for non-responsiveness and the potential inaccessibility of some farms, giving a total of 237 GPS coordinates for the study.

### Data collection

The survey was conducted between the 16th November and 1st December 2016. Data were collected using a questionnaire developed and uploaded onto the EpiCollect + mobile phone application [22]. The questionnaire included both closed and open-ended questions and was tested in the field with a limited number of smallholder farmers before implementation to make sure the questions were well understood. The main survey was conducted by five investigating teams all comprising a native Swahili speaker and paper questionnaires also available in Swahili. In every case, prior informed consent was obtained verbally from participants before interviews were conducted and after providing an overview of the aims, methodology, and anticipated outcomes of the study. Data were collected on the livestock located at the farm, farm management practices, putative risk factors for FMD, and farmers’ knowledge of the disease. An electronic version of the questionnaire is available as an Additional file 1.

To assess if farmers had experienced clinical episodes of FMD in the previous 6 months, they were asked if they had encountered cases of a disease in their livestock showing any one or more of the following clinical signs: lesions in the mouth, tongue, teats, feet, at the coronary band, and interdigital space; lameness; salivation; discharges from the nose and the mouth [23]. A farm was defined as being a case if they reported to the interview team having an animal with two or more of the clinical signs of FMD listed by AU-IBAR in the previous 6 months.

### Data analysis

Data collected from the field surveys were exported from EpiCollect + and imported into Stata 13.1 (Stata Corp, College station, Texas, USA) for data cleaning and analysis. Descriptive statistics were first calculated on the data. These included: proportions for categorical variables and means with their 95% confidence intervals (CI), medians with their interquartile range (IQR), or ranges for continuous variables. Cross tabulation was further used to summarise the data.

A spatial Bernoulli model was used to detect clustering of disease events, and estimating the relative risk of a case occurring in the predicted cluster, using SaTScan version 9.4.4 [24]. ArcGIS was used to draw maps of the study area (ESRI 2018. ArcGIS Desktop: Release 10.6 Redlands, CA: Environmental Systems Research Institute). Local spatial autocorrelation of reported FMD events was assessed by estimating the univariable Moran’s I correlation coefficient [25].

Univariable logistic regression analysis examined the associations between putative risk factors and having clinical FMD. Variables associated on univariable analysis with a *p* value less than 0.2 were taken forward into a multivariable logistic regression model. Penalized likelihood ratios were used instead of maximised likelihood ratios in the logistic regression modelling to account for low number of cases [26].

Final multivariable models were constructed using a backward-stepwise approach. Variables were included in the model based on the result of a likelihood ratio test with a *p*-value less than 0.05. Regression diagnostics were undertaken to evaluate potential multicollinearlity between independent variables by post-estimating the variance inflation factor (VIF), with model fitness assessed using the Wald χ^2^ test, Akaike’s information criterion (AIC), Bayesian information criterion (BIC), and McFadden’s Pseudo R^2^ [27]. Linearity between the continuous independent variables and the logit of the dependent variable was assessed by adding an interaction term calculated by multiplying the continuous variable with its logarithm as described before [28] and checking for its significance. Spatial autocorrelation of reported FMD cases was accounted for by including the sub-county as an a priori fixed effect.

## Results

Of the 237 GPS coordinates generated, seventeen (17/237 [7.2%]) were located either in inaccessible areas or with no discernible farm. A total of 220 small-scale dairy farmers were interviewed with an average distance between the farm and the randomly generated point of 250.8 meters (IQR 157.7–433.9). The majority of respondents were farm owners (185/220 [84.1%]) while managers and other farm workers represented the remaining 11.4% (25/220) and 4.5% (10/220), respectively. The average age of the respondents was 40.0 years (IQR 30.0–56.5).

**Figure 2:**
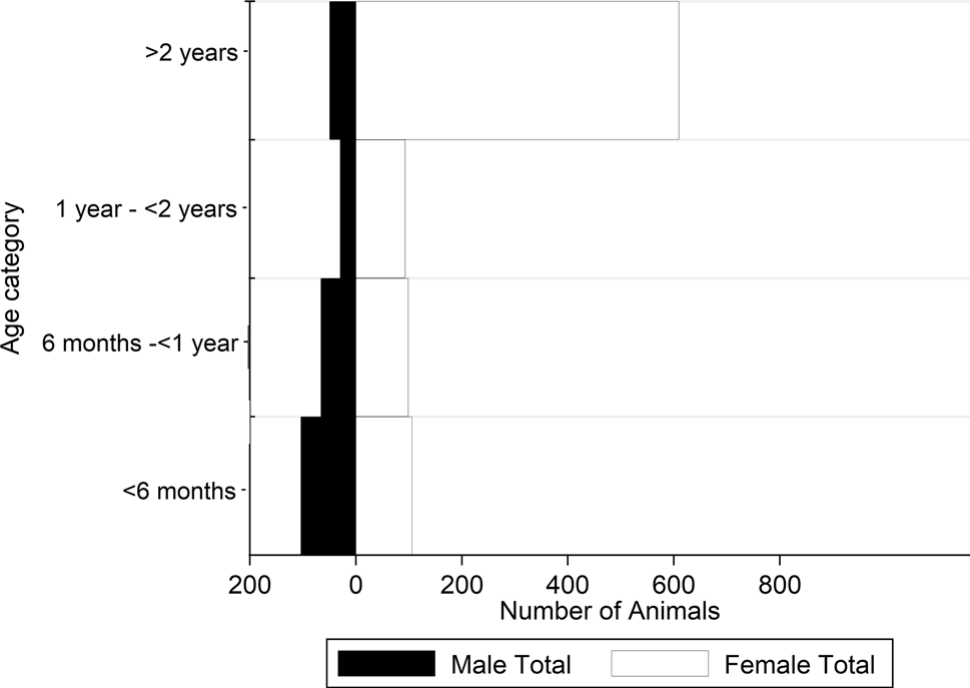
**Age distribution by sex of cattle owned by respondents.**

The surveyed farmers owned a total of 1205 cattle with the mean number of cattle kept per farmer being 4.0 (IQR 2.0–6.0). On average, more female cattle were kept across all age groups. This difference was most pronounced in cattle aged above 2 years. The majority of bulls were reported being less than 6 months of age, with their number decreasing with increasing age. The age-sex distribution of the animals under study is represented in Figure 2.

A total of 132/220 (60.0%) respondents also owned sheep, whilst 33/220 (15.0%), 22/220 (10.0%) and 1/220 (0.4%) owned goats, donkeys and pigs respectively. Twenty-four out of 220 respondents (10.9%) co-farmed both sheep and goats with cattle.

### Knowledge on FMD

The majority of farmers (207/220 [94.1%]) knew of the existence of FMD. These farmers were asked an open question on what clinical signs were typically seen in cattle affected by FMD (Table 1). The most commonly reported clinical sign was hypersalivation (160/207 [77.3%]) followed by hoof (111/207 [53.6%]) and mouth lesions (109/207 [52.7%]). Only one farmer associated the disease with mortality in adults and no farmers reported mortality in calves. Using the case definition recommended by AU-IBAR [22], 166/207 (80.2%) of the respondents who claimed knowledge of FMD correctly identified the clinical signs. A total of 70% (154/220) mentioned more than 1 clinical sign given in the case definition with 35% (77/220), 7.7% (17/220) and 0.5% (1/220) mentioning 3, 4 and all 5 signs, respectively.

**Table 1:** **Farmer knowledge of FMD clinical signs and preventive measures, including vaccination practices, in the study area located within the Nakuru County, Kenya**

Knowledge on clinical signs of FMD		Preventive measures for FMD^a^		FMD vaccination practices	
Clinical sign	Response/total^a^ (%)	Preventive measure	Response/total (%)	Vaccination practice	Response/total (%)
Hypersalivation	160/207 (77.3)	Vaccination	94/207 (45.4)	Vaccinated ≤ 4 months ago	35/220 (15.9)^b^
Hoof lesions	111/207 (53.6)	Keep cattle within farm compound	76/207 (36.7)	Vaccinated 5–6 months ago	10/220 (4.5)^b^
Mouth lesions	109/207 (52.7)	Avoid other cattle from entering farm compound	15/207 (7.2)	Vaccinated 6–12 months ago	45/220 (20.5)^b^
Lameness	81/207 (39.1)	Keep cattle away from farm compound boundaries	14/207 (6.8)	Vaccinated > 1 year ago	51/220 (31.7)^b^
Lack of appetite	64/207 (30.9)	Do not bring in new cattle	9/207 (4.3)	No vaccination date reported	2/220 (0.9)^b^
Depression	33/207 (15.9)	Avoid use of communal dips	5/207 (2.4)	Vaccinated all cattle	131/143 (91.6)^c^
Drop in milk production	15/207 (7.2)	Do not share equipment with surrounding farms	5/207 (2.4)	Young calves not vaccinated	6/143 (4.2)^c^
Lesions on teats	8/207 (3.9)	Keep visitors away from cattle	4/207 (1.9)	Pregnant Cattle not vaccinated	5/143 (3.5)^c^
Mortality in adult cattle	1/207 (0.5)	Do not do any preventive measure	58/207 (28.0)	Private AHP vaccinates cattle	24/143 (16.8)^c^
				Government AHP vaccinates cattle	118/143 (82.5)^c^
				Non-AHP vaccinates cattle	1/143 (0.7)^c^
				Cattle vaccinated at communal point	84/143 (58.7)^c^
				Cattle vaccinated at farm compound	59/143 (41.3)^c^

AHP, Animal Health Provider (veterinarian or para-veterinarian).

^a^Denominator is all farmers that had heard of FMD.

^b^Denominator is all farms that were surveyed.

^c^Denominator is all farms that had ever vaccinated.

Other vesicular diseases of cattle closely resembling FMD (such as vesicular stomatitis and bovine papular stomatitis) have not been reported in Kenya. The case definition also excluded other similar diseases occurring in the area, including Malignant Catarrhal Fever, Mucosal Disease and Bluetongue Disease.

### Reported control measures for FMD prevention

The 207 farmers who were aware of FMD were asked about preventive measures undertaken to reduce the risk of disease (Table 1). The most commonly reported preventive measure was vaccination (94/207 [45.4%]) followed by keeping cattle within the farm compound (76/207 [36.7%]). Keeping visitors away from areas on their farm compound where they might come into contact with their cattle was also reported by 4/207 farmers (1.9%). Fifty-eight respondents (28.0%) did not report any preventive measure to reduce the risk of FMD occurring on their farm.

A total of 143/207 respondents (69.1%) reported using FMD vaccine on their cattle at least once since they started farming. Farmers who reported vaccinating their animals in the previous 4 and 6 months were 35/207 (16.9%) and 45/207 (21.7%), respectively.

### FMD 6-month occurrence

Of the total 220 smallholder farmers, 13 (5.9% [95% CI 2.8–9.0]) reported having a case of FMD in at least one animal on their farm in the previous 6 months, all correctly identifying the disease according to the AU-IBAR case definition. Of these, 84.6% (11/13) mentioned more than two clinical signs given in the case definition. A total of 53.8% (7/13), 23.1% (3/13) and 7.7% (1/13) mentioned 3, 4 and all 5 clinical signs in the case definition, respectively. When the number of clinical signs reported by respondents was modelled against whether a farm reported a case of FMD, the probability of reporting a clear case of FMD increased by 1.9 (*p* = 0.027) when respondents provided an additional clinical feature of the disease.

Of these farms, 60 individual cases of FMD were reported representing an individual level incidence risk of 5.0% (95% CI 3.9–6.4) based on the 1205 cattle owned by surveyed farms at the time of the survey. Based on the estimated numbers of cattle present at the time of the outbreak (mean number per affected farm of 8.2, 95% CI 2.9–13.6) and the numbers affected with FMD (mean number of cases per affected farm of 4.6, 95% CI 0.8–8.5), the mean within-farm incidence risk was 58.0% (95% CI 38.3–77.6).

**Figure 3:**
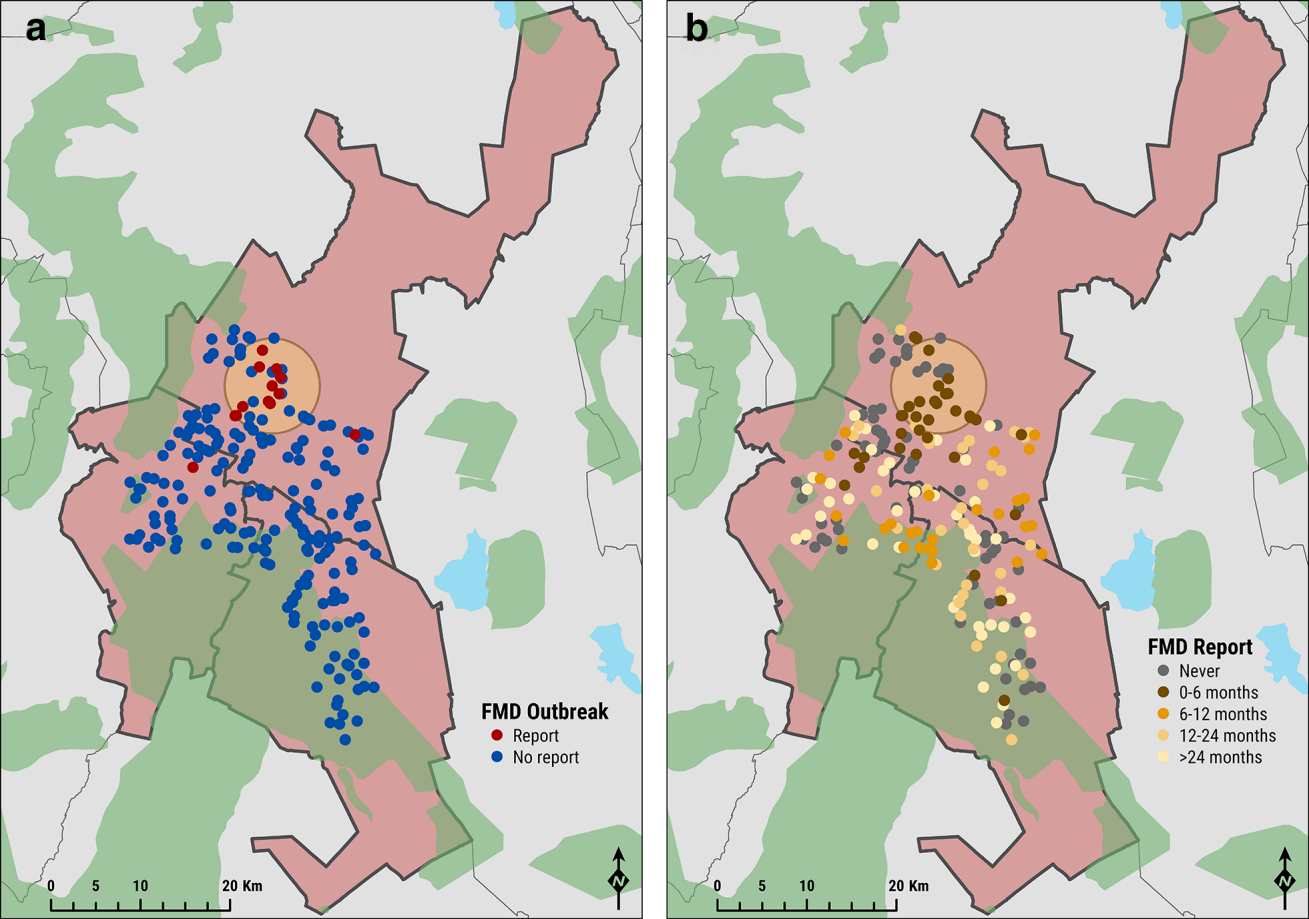
**Map of occurrence of FMD cases.** Farmer reported FMD outbreak locations within the study area are represented in red in **A**, whilst the distribution of farmer reported FMD occurrence reported during the survey is reported in **B**. The geographical cluster of cases is also shown (light orange colour).

Spatial clustering of farms reporting FMD cases in the previous 6 months was identified in the northern part of the study area (Northern part of Rongai), observing a significant non-zero positive spatial autocorrelation between cases (Moran’s I = 0.508; *z* = 7.084; *p *≤ 0.001) (Figure 3). This single disease cluster (with an estimated radius of 5.32 km) was 38.1 times more likely than any other part of the study area to experience FMD (*p* < 0.001), with 40.7% of the clinical cases reported within the geographical extent of this cluster.

### Retrospective history of FMD occurrence within the surveyed area

Farmers were asked when they last heard of an FMD outbreak in the local area. Of the 220 respondents, 25/220 (11.4%) claimed never to have heard of an FMD case in their local area. For farmers that had heard of FMD in the local area, 34/220 (15.5%) stated FMD was reported in the previous 6 months, 29/220 (13.2%) between 6 months and 1 year, 33/220 (15.0%) between 1 and 2 years, and 45/220 (20.5%) more than 2 years ago. A total of 54/220 (24.6%) farmers did not respond to the question although the reasons for non-response were not included.

### Reports of wildlife in the area

The majority of farmers (130/220 [59.0%]) had not seen or heard of reports of FMD susceptible wildlife in the surrounding areas and outside of the parks. A total of 21 out of 220 (9.5%) heard of reports of antelopes, 1/220 (0.5%) of gazelles and 1/220 (0.5%) of wild pigs. Other wildlife not naturally susceptible to FMD reported by these respondents included aardvarks, cheetahs, hyena, hares, leopards, monkeys, porcupines, squirrels, and wild dogs.

### Farm level risk factors for FMD occurrence

Putative risk factors for FMD among smallholder farmers in the study area are shown in Table 1. Most farmers used artificial insemination (AI) (115/220 [52.3%]) to breed their cattle. This consisted of 96/220 (43.6%) who used AI solely, 2/220 (0.9%) who used AI together with their own bull, and 17/220 (7.7%) who used AI together with a shared bull. A total of 67/220 (30.5%) reported using a shared bull, subdivided further into 46/220 (20.9%) who used a shared bull only and 4/220 (1.8%) using both shared and their own bull.

A total of 89/220 (40.5%) had acquired new cattle in the previous 12 months. Of these 89 respondents, 54 (60.7%) had acquired only one replacement animal in the previous year, with the rest acquiring two or more. For sourcing new cattle, 96/220 (43.6%) used surrounding farms while 43/220 (19.6%) used livestock markets.

Less than half of farmers (97/220 [44.1%]) used communal grazing either as the sole source of pasture (34/220 [15.5%]) or in addition to that available within the farm compound (63/220 [28.6%]). For the 97 respondents that used communal grazing, roadsides were the most commonly used (68/97 [70.1%]), while 58/97 (59.8%) used other non-questionnaire listed communal places (e.g. harvested fields), 10/97 (10.3%) forests and 9/97 (9.3%) used fields within towns. Communal watering points for cattle were used by 64/220 (29.1%) of the farmers interviewed, whilst communal acaricide dips were used by 14/220 (6.4%).

Several factors were associated with an increased risk of clinical FMD in the previous 6 months based on univariable analysis, including: use of a shared bull; the number of additional cattle sourced from outside the farm in the previous 12 months; buying cattle from livestock markets; grazing sheep both within towns and the farm compound; grazing cattle within towns; use of a communal dip; and the number of sheep present on farm. The odds of disease being reported was significantly lower in farms that had used vaccination at some point in their past (OR = 0.2, 95% CI 0.07–0.7; *p* = 0.013). The results of the univariable analysis for all putative risk factors are shown in Additional file 2.

The final multivariable model contained an a priori term (subcounty) to correct for spatial autocorrelation (Table 2). Based on this final multivariable model, the use of a shared bull was significantly associated with the FMD status of a farm (OR = 9.7, 95% CI 1.6–59.1; *p* = 0.014) when compared to those not using this breeding method. A number of farmers (49/220) did not respond to the question on breeding method. These were included as a separate category and there was no evidence of an association with reporting FMD (OR = 3.4 (95% CI 0.4–25.1; *p* = 0.238). Due to collinearity with the variable representing the use of a shared bull, the use of AI was dropped as a separate variable in the model. The odds of FMD increased 1.1 times for each additional sheep owned (95% CI 1.0–1.2; *p* = 0.066). The interaction term for the number of sheep and its logarithm in the final multivariable model was not significant (*p* = 0.251) indicating that linearity with the logit of the dependent variable was not violated.

**Table 2:** **Odds ratios from logistic regression indicating associations between exposure variables and the odds of having FMD in the previous 6** **months**

Univariable analysis				Multivariable analysis	
Variable	Type of variable	Odds ratio (95% CI)	p value	Odds ratio (95% CI)	p value
Use of a shared bull	Categorical				
Did not use a shared bull		Base category		Base category	
Used a shared bull		12.7 (0.4–396.4)	0.147	9.7 (1.6–59.1)	0.014
Did not respond to the question		15.4 (2.1–112.5)	0.007	3.4 (0.4–25.1)	0.238
Number of additional cattle sourced from outside the farm in the previous 12 months	Continuous	1.2 (1.0–1.5)	0.043	1.1 (1.0–1.3)	0.207
Buying cattle from livestock markets in the previous 12 months	Categorical	3.9 (1.3–12.4)	0.019		
Grazing sheep within towns	Categorical	34.5 (3.5–337.8)	0.002		
Grazing cattle within towns	Categorical	8.3 (1.6–43.4)	0.012		
Use of communal dips	Categorical	8.6 (2.3–32.8)	0.002		
Number of sheep	Continuous	1.1 (1.0–1.2)	0.025	1.1 (1.0–1.2)	0.066
Ever vaccinated cattle for FMD	Categorical	0.2 (0.07–0.7)	0.013		
Subcounty	Categorical				
Njoro		Base category		Base category	
Molo		5.3 (0.2–133.5)	0.307	4.5 (0.2–113.6)	0.365
Rongai		37.4 (2.2–643.2)	0.013	30.0 (1.7–528.9)	0.020

From the univariable analysis, only variables with a *p*-value < 0.2 are included (a list of all examined variables is reported in Additional file 1) which were taken forward into the final multivariable model using a backward-stepwise approach. Subcounty of the interviewed farm was included a priori as a fixed effect to account for potential spatial autocorrelation. Variables were retained in the final model if the likelihood ratio test had a *p*-value less than 0.2. The final multivariable model had a Wald Chi square of 20.0 with 6 degrees of freedom giving a *p* value of 0.0027. The model had an AIC of 64.5, a BIC of 95.0 and a McFadden’s R^2^ of 0.443.

## Discussion

Foot-and-mouth disease has major economic implications to dairy farming systems in Kenya and other developing countries within the African continent [2]. Despite various studies ranking FMD among the most important animal diseases among cattle keepers in Kenya [4, 29–32], no other study has aimed to determine the knowledge, attitudes and practices towards it among small-holder dairy farmers communities.

The number of cattle kept on the farms surveyed in this study were similar to other smallholder studies from the region [33–35] and, in addition to keeping cattle, the small-scale farmers interviewed also kept sheep, goats and pigs which are susceptible to FMD. This diversification of livestock was also reported by Njarui et al. [34] in a study conducted in the highland counties of Kenya. The same finding was also reported by Kosgey et al. [36] in an earlier study in Nakuru, Nandi and Nyeri Counties of Kenya. In Kenya and other African countries small stock are kept as a quick source of liquidity in the face of family needs such as school fees and payment of dowry [37]. The age-sex distribution in the study population was consistent with the dominance of dairy production systems in this region, with high numbers of adult females and a gradual decrease in numbers of males with increasing age indicating likely retention for breeding purposes.

By using random spatial sampling, it was possible that farms in high density areas might have had a lower probability of being selected than those in low density areas. The authors accept that this bias potentially exists although in the absence of a sampling frame and with a population census several years out of date, this was considered the optimal approach with the resources available. It was assumed that small scale dairy farmers were evenly distributed in the study area. However, the geographical extent of the study area was not large and the author’s knowledge of the study area would suggest that this is a reasonable assumption and the potential for bias was limited.

The survey results revealed that the majority of farmers in the study population had knowledge of FMD. The most commonly reported clinical sign was hypersalivation, followed by hoof and mouth lesions. Based on the FMD case definition recommended by the AU-IBAR [23], the majority of respondents correctly identified the disease providing some internal validity to the study results. Only one respondent reported observing mortality due to FMD, which was in adult cattle. No farmer reported mortality among calves. This is consistent with a SAT 2 outbreak on a large-scale farm in the study area in 2012 that reported a mortality rate of 0.44%, reflecting a single adult death related to FMD [15]. FMD is often associated with deaths among young stock from myocardial infection [38]. In neighbouring Ethiopia, a study reported the highest mortality among cattle less than 2 years of age at 2.8% [39]. Reasons for the low mortality in the present study may be attributable to farmers associating sudden deaths with other diseases, different pathogenicity of the circulating strains, or the presence of maternal immunity associated with previous exposure and vaccination.

Vaccination was the most frequently reported preventive measure against FMD, followed by restricting contact with other cattle by keeping them within the farm compound. Nevertheless, nearly a third of respondents reported doing nothing to prevent FMD from occurring in their livestock, although it is unclear if this is due to a lack of knowledge, a perceived low risk of disease, or difficulties achieving recommended preventive measures. Follow-up studies are required to explore this observation and may indicate a requirement for public awareness and education programmes on FMD prevention among farmers in this region.

The only FMD vaccine available at the time of the study was an aqueous-adjuvanted, inactivated vaccine with a recommended vaccination interval of 6 months [40]. Despite vaccination being the most reported preventive measure (45%), the estimated vaccination coverage for the last 6 months was lower (21.7%). This may indicate either a lack of knowledge over the necessary vaccination schedules or poor vaccine availability. However, the percentage of farms that had ever vaccinated was markedly higher (69.1%) than those stating vaccination was used to prevent FMD. This disparity may indicate that some farmers were unaware of the purpose of vaccination. Quantifying and deploying effective vaccination coverage at a population level is an essential component of any FMD control programme in an endemic setting. Uncertainty in vaccination coverage estimates could be addressed through improved record keeping including the use of vaccination record cards as recommended in the FAO-OIE Post Vaccination Monitoring Guidelines [41]. Some farmers reported not vaccinating young and pregnant cattle. Young calves are often not included in vaccination campaigns due to the presence of maternal antibodies that can interfere with the immune response. There may also be a perception that the impact of disease is lower among this group leading to reluctance to pay for vaccination. The lower vaccination among pregnant cattle may be due to an association with pre-term calving or abortion. Further studies and subsequent public awareness programs would be useful in educating farmers on recommended vaccination practices.

Spatial clustering of FMD affected farms was identified within the Rongai sub-county, which may indicate a geographical structure of FMD circulation. Identifying clinical disease clusters is useful for informing a risk-based control strategy by targeting control measures to these areas. The clustering observed in this study is likely to be attributable to a transit route for pastoralists in this area [39]. Although pastoralist routes within the study area have not been mapped, many farmers suggested that the occurrence of FMD coincided with the arrival of Maasai pastoralists to utilise available grazing.

The proportion of farmers that reported having heard of FMD in the study area 6 months prior to the survey was 15.5% compared to 5.9% that reported having disease. This means that more farmers had heard of outbreaks in their area than those that actually experienced a case in their farm, perhaps because they had not received information on an outbreak being reported in the area. Although data were not collected on how disease information was conveyed, this finding suggests that communication of outbreaks could be improved so that farmers could initiate preventative measures. This could be achieved through public awareness campaigns, mobile phone messaging or social media. The prevalence estimate in the present study was less than the expected prevalence used for sampling size calculation, and the results from a previous serological survey of the area [3]. The expected prevalence was based on a limited number of respondents usually interviewed during EuFMD training activities in the county, thus potentially not providing enough power and also bias as these studies were performed in areas of known FMD virus circulation. It was indeed lower than that estimated by serology and this difference can be explained by the fact that seroprevalence reports levels of lifetime exposure to the virus. In addition, the present study investigated the presence of clinical disease which may not correlate with seropositivity.

The present study used clinical signs for the case definition with no laboratory confirmation. There are limitations to this approach although there was some validation through comparing reported clinical signs to the AU-IBAR case definition [23]. However, because of the imperfectness of our case definition methodology (specificity and sensitivity is unlikely to be close to 1), both the FMD occurrence and FMD odds here reported are likely to be biased estimators of the true FMD status in the area 6 months prior to the study [42]. In a cross sectional study in Cambodia, Bellet et al. [43] compared participatory epidemiology tools (including farmer description of clinical signs of FMD) with serological tests. The authors found participatory methods as characterised by high sensitivity and low specificity in the identification of FMD cases. To overcome this, serosurveys could be useful. However, serosurveys used to estimate the burden of infection can be time-consuming and expensive. Moreover it is difficult to estimate the timing of infection as antibody levels can persist for years post-infection [44] and previous vaccination complicates interpretation particularly if not using vaccines that have been specifically purified of non-structural proteins [13]. Surveys for clinical disease offer a low-cost alternative that is likely to be more achievable in resource poor settings, although these do not replace the need for serosurveys in understanding the epidemiology of FMD.

The majority of farmers used AI for breeding their livestock (52.3%). This is higher than that reported by Baltenweck et al. [45] who found only 18.6% of the smallholder dairy farmers in Kirinyaga, Nakuru and Kisumu Counties in Kenya using this breeding method. This high figure may be due to an increased accessibility to AI services. Temporal changes in management practices may be related to a dynamic risk of FMD exposure and affect the impact of risk-based control measures.

Using communal resources for grazing and water was commonly reported in this study and consistent with other studies in Kenya [39, 40]. Farmers often resort to communal sources during the dry seasons when grazing and water are scarce, increasing the potential for transmission of infectious pathogens like FMDV. Despite many farms using communal grazing and water, neither was associated with the occurrence of FMD in this study. This may be related to the timing of the study (November, with the main dry season running from January through to March) since farmers were asked if clinical FMD had occurred in the previous 6 months. This may indicate that using communal resources are relatively lower risk outside this dry season although further studies are required to investigate this hypothesis. Communal acaricide dips are another potential cause of livestock contact and are used all year around. Despite their use being associated with clinical FMD on univariable analysis, this variable was dropped from the multivariable model. Relatively few farmers (6.4%) used communal dips for tick control so the study is likely to be underpowered to show an association if present.

Contact with FMDV susceptible wild animals is a potential risk factor for disease [46]. Farmers reported the presence of antelopes and wild pigs in the surrounding areas although the presence of wildlife was not a significant risk factor in this study. This result is not surprising since the majority of small scale farmers in Kenya do not graze animals in protected areas where they might interact with wildlife [47]. In addition, Lake Nakuru National Park is fenced so likely reducing the probability of contact [48], confirmed by the minimal sightings of wildlife in the study area.

Several risk factors for FMD were identified by univariable analyses at the farm level while only vaccination was associated with a lower risk of disease. This is in agreement with studies conducted elsewhere on similar and differing settings [10–12, 49]. Vaccination was not associated with a reduced disease risk in the multivariable model so it is likely that there was confounding with the univariable association. However, this study was not designed to evaluate vaccine effectiveness, therefore no reliable assessment of vaccine performance can be made.

Multivariable analysis indicated that the use of a shared bull was related to FMD occurrence on the farm in the previous 6 months. Shared bulls present a high risk for moving between farms and having contact with potentially infected animals. Forty-nine (49/220, 22.3%) farmers did not report any breeding method. There was no evidence that these farmers were at greater or lesser risk of having reported clinical disease in the multivariable model. It is possible that the reason for these farmers not reporting a breeding method was that they did not breed their cattle, although this information was not recorded. For every sheep owned by a farm the odds of introducing FMD increased by 10%. This finding agrees with Mergesa et al. [9] who identified co-farming cattle with small ruminants as a risk factor for FMD in pastoralist systems in Ethiopia, although they did not investigate the effect of the number of small ruminants. In a study by Anderson et al. [50] on the role of sheep and goats in FMD epidemiology in Kenya, a high seropositivity level was reported thus indicating likely exposure in small ruminants. Observations from the study area indicated that mixed cattle-small ruminant farms were often managed differently to farms that only kept cattle. This may include factors that increase the risk of exposure to FMD virus in small ruminants (e.g. communal grazing over wider areas and for longer periods), which could be transmitted to cattle where disease is more apparent. Small ruminants are commonly excluded from vaccination strategies (including Kenya) though their inclusion could be beneficial by reducing interspecies transmission. Although challenge studies have indicated a limited role for sheep in FMD transmission to cattle [51], further evidence derived from field conditions are required to support their inclusion in vaccination strategies.

In conclusion, FMD is regularly reported among smallholder dairy farmers in Nakuru County, Kenya, which in this study affected 1 in 17 farms over a six-month period. Farmers had knowledge of FMD and the associated clinical signs, but the disease control by vaccination and its coverage reported in this area was low. There is a need to educate farmers on the risk of FMD and associated control measures including vaccination, enhancing their access. Improved understanding of FMD epidemiology can help identify risk-based control measures that can be implemented to reduce disease impact. Use of shared bulls and co-farming sheep with cattle were identified as risk factors for disease in this study. Although semi-structured questionnaire-based surveys have limitations, the current study shows that useful information on the burden of disease can be easily extracted from rural farming communities in low resource settings.

## Additional files

**Additional file 1.** **Questionnaire for dairy farmers.** This file contains the paper form of the questionnaire tha was used in the data collection for the study.

**Additional file 2.** **Table showing the results of univariable analysis of all putative risk factors against each investigated variable.** This file contains the results of univariable logistic regression carried out on the relevant study variables against an outcome of whether or not a farm experienced a case of FMD.

## Data Availability

The dataset supporting the conclusions of this article is available in the Harvard Dataserve repository: 10.7910/DVN/KQMKPZ.

## Abbreviations

AIC: akaike information criterion
AI: artificial insemination
AU-IBAR: African Union Intergovernmental Bureau of Animal Resources
BIC: Bayesian Information Criterion
EUFMD: European Union Commission for the control of Foot and Mouth Disease
FMD: foot and mouth disease
FMDV: foot and mouth disease virus
PCP: progressive control pathway
PD_50_: protective dose 50
SAT 1: Southern African Territories 1
SAT 2: Southern African Territories 2
VIF: variance inflation factor
