# Supplementary material for: Knowledge and risk factors for foot-and-mouth disease among small-scale dairy farmers in an endemic setting
Source: Vet Res. 2019 May 14;50:33. doi: 10.1186/s13567-019-0652-0 (PMC6518695; doi:10.1186/s13567-019-0652-0)
Supplement: Supplementary file 1 — Additional file 1. Questionnaire for dairy farmers. This file contains the paper form of the questionnaire tha was used in the data collection for the study. [file 13567_2019_652_MOESM1_ESM.docx]

| Title | Risk factor | Category | N (Col %) | Percentage with FMD | Odds ratio (95% CI) | *p*-value |
| --- | --- | --- | --- | --- | --- | --- |
| Breeding method | Use of a shared bull | Yes | 67/220 (30.5) | 9/67 (13.4) | 11.21 (1.94-64.58) | 0.007 |
|  |  | No | 104/220 (47.3) | 1/104 (1.0) | Base category |  |
|  |  | No response | 49/220 (22.3) | 3/49 (6.1) | 5.19 (0.74 – 36.28) | 0.097 |
|  | Artificial Insemination | Yes | 115/220 (52.3) | 4/115 (3.5) | 0.31 (0.09-1.09) | 0.068 |
|  |  | No | 56/220 (25.45) | 6/105 (5.7) | Base category |  |
|  |  | No response | 49/220 (22.27) | 3/49 (6.12) | 0.58 (0.15 – 2.28) | 0.439 |
|  | Number of replacement cattle purchased in the last 1 year. | Continuous variable | | | 1.21 (1.03-1.42) | 0.022 |
| Source of replacement stock | Source of replacement is own animals | Yes | 147/220 (66.8) | 6/147 (4.1) | 0.41 (0.14-1.21) | 0.106 |
|  |  | No | 73/220 (33.2) | 7/73 (9.6) | Base category |  |
|  | Buying replacement from surrounding farms | Yes | 96/220 (43.6) | 6/96 (6.3) | 1.13 (0.38-3.33) | 0.831 |
|  |  | No | 124/220 (56.4) | 7/124 (5.7) | Base category |  |
|  | Buying replacement animals from livestock markets | yes | 43/220 (19.6) | 6/43 (14.0) | 3.94 (1.30-11.94) | 0.015 |
|  |  | No | 177/220 (80.4) | 7/177 (4.0) | Base category |  |
| Goat grazing locations | Goats grazing in other communal places | Yes | 11/220 (5.0) | 1/11 (9.1) | 3.27 (0.39-27.49) | 0.276 |
|  |  | No | 10/220 (4.55) | 3/10 (30) | Base category |  |
|  |  | Did not keep goats | 199/220 (90.45) | 9/199 (4.5) | 0.35 (0.06 – 2.18) | 0.260 |
|  | Goats grazing by road sides | Yes | 7/220 (3.18) | 1/7 (14.3) | 0.76 (0.09-6.45) | 0.800 |
|  |  | No | 14/220 (6.36) | 3/14 (21.4) | Base category |  |
|  |  | Did not keep goats | 199/220 (90.45) | 9/199 (4.52) | 0.16 (0.04 – 0.64) | 0.009 |
|  | Goats grazing within towns | Yes | 2/220 (0.9) | 1/ 2 (50) | 4.71 (0.37-59.78) | 0.232 |
|  |  | No | 19/220 (8.64) | 3/19 (15.8) | Base Category |  |
|  |  | Did not keep goats | 199/220 (90.45) | 9/199 (4.52) | 0.24 (0.06 – 0.88) | 0.032 |
|  | Grazing goats in forests | Yes | 2/220 (0.91) | 1 /2 (50) | 4.71 (0.37-59.78) | 0.232 |
|  |  | No | 19/220 (8.64) | 3/19 (15.8) | Base category |  |
|  |  | Did not keep goats | 199/220 (90.45) | 9/199 (4.52) | 0.24 (0.06 – 0.88) | 0.032 |
| Goat grazing methods | Grazing goats within the compound | Yes | 27/220 (12.27) | 3/27 (11.1) | 0.52 (0.06-4.38) | 0.551 |
|  |  | No | 6/220 (2.73) | 1/6 (16.7) | Base category |  |
|  |  | Did not keep goats | 187/220 (85) | 9/187 (4.81) | 0.20 (0.03 – 1.33) | 0.095 |
|  | Grazing goats outside the compound | Yes | 17/33 (51.5) | 4/17 (23.5) | 6.09 (1.65-22.45) | 0.007 |
|  |  | No | 16/33 (48.5) | 0/16 (0) | Base category |  |
| Sheep graze locations | Grazing sheep by road sides | Yes | 37/220 (16.82) | 3/37 (8.1) | 0.62 (0.09-4.11) | 0.618 |
|  |  | No | 16/220 (7.27) | 2/16 (12.5) | Base category |  |
|  |  | Did not keep sheep | 167/220 (75.91) | 8/167 (4.79) | 0.31 (0.07 – 1.40) | 0.127 |
|  | Grazing sheep within towns | Yes | 5/220 (2.27) | 3/5 (60) | 26.04 (3.31-204.79) | 0.002 |
|  |  | No | 48/220 (21.82) | 2/48 (4.2) | Base Category |  |
|  |  | Did not keep sheep | 167/220 (75.91) | 8/167 (4.79) | 0.99 (0.23 – 4.22) | 0.990 |
| Sheep grazing methods | Grazing sheep outside compound | Yes | 62/220 (28.18) | 6/62 (9.7) | 2.28 (0.59-8.78) | 0.229 |
|  |  | No | 72/220 (32.73) | 3/72 (4.2) | Base category |  |
|  |  | Did not keep sheep | 86/220 (39.09) | 4/86 (4.65) | 1.08 (0.26 – 4.54) | 0.913 |
|  | Grazing sheep within compound | Yes | 103/220 (46.82) | 7/103 (6.8) | 0.91 (0.21-4.07) | 0.909 |
|  |  | No | 31/220 (14.09) | 2/31 (6.5) | Base category |  |
|  |  | Did not keep sheep | 86/220 (39.09) | 4/86 (4.65) | 0.64 (0.13 – 3.19) | 0.590 |
|  | How often cattle grazed on communal grounds | Always | 55/220 (25) | 5/55 (9.1) | 2.46 (0.68-8.93) | 0.172 |
|  |  | Sometimes | 46/220 (20.9) | 3/46 (6.5) | 1.81 (0.43 – 7.67) | 0.418 |
|  |  | Rarely | 14/220 (6.4) | 1/14 (7.1) | 2.51 (0.36-17.32) | 0.352 |
|  |  | Never | 105/220 (47.7) | 4/105 (3.8) | Base category |  |
| Cattle grazing locations | Grazing cattle in other communal places | Yes | 58/220 (26.36) | 6/58 (10.3) | 1.86 (0.41-8.47) | 0.424 |
|  |  | No | 39/220 (17.73) | 2/39 (5.1) | Base category |  |
|  |  | No response | 123/220 (55.91) | 5/123 (4.07) | 0.70 (0.15 – 3.25) | 0.645 |
|  | Grazing cattle by road sides | Yes | 68/97 (70.1) | 6/68 (8.8) | 1.14 (0.25-5.26) | 0.863 |
|  |  | No | 29/97 (29.9) | 2/29 (6.9) | Base category |  |
|  |  | No response | 123/220 (55.91) | 5/123 (4.07) | 0.51 (0.11 – 2.41) | 0.396 |
|  | Grazing cattle within towns | Yes | 9/220 (4.1) | 3/9 (33.3) | 8.17 (1.72 -38.90) | 0.008 |
|  |  | No | 88/220 (40.0) | 5/211 (2.4) | Base category |  |
|  |  | No response | 123/220 (55.91) | 5/123 (4.07) | 0.70 (0.21 – 2.37) | 0.572 |
| Cattle grazing methods | Grazing cattle outside compound | Yes | 100/220 (45.4) | 9/100 (9.0) | 2.69 (0.85-8.53) | 0.093 |
|  |  | No | 120/220 (54.6) | 4/120 (3.3) | Base category |  |
|  | Grazing cattle within compound | Yes | 171/220 (77.7) | 11/171 (6.43) | 1.36 (0.33-5.55) | 0.667 |
|  |  | No | 49/220 (22.3) | 2/49 (4.1) | Base category |  |
|  | Vaccination against FMD | Yes | 143/220 (65.0) | 5/143 (3.5) | 0.23 (0.07-0.74) | 0.013 |
|  |  | No | 77/220 (35.0) | 8/77 (10.4) | Base category |  |
|  | The farm is next to a road | Yes | 205/220 (93.2) | 12/105 (5.8) | 1.60 (0.27-9.46) | 0.603 |
|  |  | No | 15/220 (6.8) | 1/15 (6.7) |  |  |
|  | Sharing of equipment with other farms | Yes | 66/220 (30.0) | 5/66 (7.6) | Base category |  |
|  |  | No | 154/220 (70) | 8/154 (5.2) | 0.65 (0.21-1.97) | 0.446 |
|  | Employees commute from outside farm | Yes | 28/220 (12.73) | 3/28 (10.7) | 2.37 (0.66-8.52) | 0.185 |
|  |  | No | 191/220 (86.82) | 10/191 (5.24) | Base category |  |
|  |  | No response | 1/220 (0.45) | 0/1 (0) | 5.76 (0.22 – 150.17) | 0.292 |
|  | Sharing of workers with other farms | Yes | 26/220 (11.8) | 3/26 (11.5) | 2.62 (0.72-9.45) | 0.142 |
|  |  | No | 194/220 (88.2) | 10/194 (5.2) | Base category |  |
|  | Use of communal dips | Yes | 14/220 (6.4) | 4/14 (28.6) | 8.77 (2.43-31.62) | 0.001 |
|  |  | No | 203/220 (92.27) | 9/206 (4.4) | Base category |  |
|  |  | No response | 3/220 (1.36) | 0/220 (0) | 2.92 (0.14 – 60.77) | 0.488 |
|  | Use of communal watering holes | Yes | 64/220 (29.1) | 6/64 (9.4%) | 2.21 (0.74-6.61) | 0.154 |
|  |  | No | 156/220 (70.9) | 7/156 (4.5) | Base category |  |
|  | How often goats grazed on communal grounds | Always | 13/33 (39.4) | 1/13 (7.7) | 1.32 (0.12-14.26) | 0.819 |
|  |  | Sometimes | 2/33 (6.1) | 1/ 2 (50) | 11 (0.66 – 183.35) | 0.095 |
|  |  | Rarely | 1/33 (3.0) | 1 (100) | 33 (0.89 – 1220.82) | 0.058 |
|  |  | Never | 17/33 (51.5) | 1/17 (5.9) | Base category |  |
|  | Number of goats | Continuous Variable | | | 1.06 (0.93-1.22) | 0.384 |
|  | Owning of goats in addition to cattle | Yes | 33/220 (15.0) | 4/33 (12.1) | 2.87 (0.87-9.40) | 0.082 |
|  |  | No | 187/220 (85.0) | 9/187 (4.8) | Base category |  |
|  | How often sheep grazed on communal grounds | Always | 41/132 (31.1) | 3/41 (7.3) | 1.36 (0.32-5.82) | 0.42 |
|  |  | Sometimes | 17/132 (12.9) | 1/17 (5.9) | 1.34 (0.20 – 9.34) | 0.32 |
|  |  | Rarely | 3/132 (2.3) | 1/3 (33.3) | 9.0 (0.96 – 84.36) | 0.054 |
|  |  | Never | 71/132 (53.8) | 4/71 (5.6) | Base category |  |
|  | Number of sheep owned | Continuous variable | | | 1.08 (1.02-1.16) | 0.016 |
|  | Owning of sheep | Yes | 132/220 (60.0) | 9/132 (6.8) | 1.44 (0.45-4.59) | 0.533 |
|  |  | No | 88/220 (40.0) | 4/88 (4.6) | Base category |  |
|  | The number of cattle below 6 months owned | Continuous variable | | | 1.18 (0.86-1.63) | 0.304 |
