# Supplementary material for: Knowledge and risk factors for foot-and-mouth disease among small-scale dairy farmers in an endemic setting
Source: Vet Res. 2019 May 14;50:33. doi: 10.1186/s13567-019-0652-0 (PMC6518695; doi:10.1186/s13567-019-0652-0)
Supplement: Supplementary file 2 — Additional file 2. Table showing the results of univariable analysis of all putative risk factors against each investigated variable. This file contains the results of univariable logistic regression carried out on the relevant study variables against an outcome of whether or not a farm experienced a case of FMD. [file 13567_2019_652_MOESM2_ESM.pdf]

Additional File 2

Questionnaire for dairy farmers

Farm ID: **Date:**

MINISTRY OF AGRICULTURE LIVESTOCK AND FISHERIES
DIRECTORATE OF VETERINARY SERVICES

Survey Questionnaire: BSv1.6

Prevalence and Risk Factors for Foot-and-Mouth Disease in Small Scale Dairy Farmers in Nakuru County and its Environs

Baseline Data

1. Position at the farm: (please circle the correct choice)
    a. Owner
    b. Employee
    c. Milker
    d. Herdsman
    e. Manager
    f. Other ____

2. GPS coordinates: longitude____________ latitude______________

3. Age in years: ________________

4. Level of education?
    a. None
    b. Primary
    c. Secondary
    d. Certificate
    e. Diploma
    f. Degree
    g. Post graduate

5. How many years have you been doing dairy farming? ______________
6. What is the size of farm (where the cattle are) in acres? _______________
7. What other species are found in your farm (state number):
    a. Goats _________
    b. Sheep__________________
    c. Pigs___________

    d. Donkeys________

8. How many cattle are currently on the farm? _______________
   Please complete the following grid to give the sex and age categories for cattle on the farm

Age of cow	Male	Female
< 6 months		
6-12 months		
1-2 years		
>2 years		

Risk factors

9. What grazing methods do you use for your animals?
   a. Stall feeding
   b. Semi grazing (Animals allowed to graze but additional food is given by stall feeding)
   c. Pasture grazing only
10. Do you use communal grazing fields to feed your animals?
    a. Yes
    b. No
    c. Don't know

Please complete table showing how grazing varies for each species:

Species	Grazing method<br>1 = Zero; 2 = semi grazing; 3 = pasture only	Communal grazing?<br>Y=Yes; N=No
Cattle		
Sheep		
Goat		
Pigs		

11. Do you use communal watering facilities to water your animals?
    a. Yes
    b. No
12. If you do semi or full time grazing, do you ever graze your animals in (yes or No):
    a. Forests___________
    b. Within towns___________
    c. By roadsides_________
    d. Near livestock markets or slaughterhouses_________
13. Do you buy unprocessed animal feeds (hay, grass etc.) from outside your farm?

a. Yes
b. No

14. Do you share workers with other surrounding farms?
    a. Yes
    b. No
    c. Don’t know
15. Do employees commute from outside the farm?
    a. Yes (go to question 16)
    b. No (go to question 17)
    c. Don’t know (go to question 17)
16. If yes to the question above, do any of the commuting workers own animals?
    a. Yes
    b. No
    c. Don’t know
17. Do you share equipment with surrounding farms?
    a. Yes
    b. No
    c. Don’t Know
18. Is your farm next to a road (where animals and/or people pass through)?
    a. Yes
    b. No
19. In the surrounding areas have there been reports of presence of buffaloes, antelopes, wild pigs or other wild animals? Please complete grid,

**Species**	**Yes/ No**
Buffalo	
Antelope	
Wild pigs	
Other (please state)	

20. Where do you get your replacement stock?
    a. Buying from cattle markets
    b. Buying from surrounding farms
    c. From my own animals
    d. Other (please state)
21. If you buy your replacement stock, what number of cattle did you buy in the last 1 year? ________

Knowledge and occurrence of FMD

22. Have you heard of foot and mouth disease?
    a. Yes (go to question 23)
    b. No (go to question 24)
23. What signs do you see in animals affected with foot-and-mouth disease?
    a. ____
    b. ________________________
    c. ________________________
    d. ____
    e. ________________________

24. Do you call a veterinarian/animal health assistant when there is a case of foot and mouth disease on your farm?
    a. Yes
    b. No
    c. Don't know
25. When there is a foot and mouth disease case do you report it to the veterinary authorities?
    a. Yes
    b. No
    c. Don't know
26. Have you had any cases of foot and mouth disease in the farm in the last 6 months?
    a. Yes
    b. No
    c. Don't know
27. When did you last have a case of FMD in your farm? (Month/year)
    a. ________________/__________________
    b. Can't remember
28. How many animals did you have at the time (if don't know, write "DK")? ____________
29. How many animals were affected (if don't know, write "DK")? __________
30. Were the affected animals being milked at the time?
    a. Yes (If yes, how many____________)
    b. No
    c. Don't know

31. Of the animals affected how many were:

	**Male**	**Female**
< 6 months		
6-12 months		
1-2 years		
>2 years		

32. When was the last time you heard of any foot and mouth disease case in your village? _____________weeks/months/years ago (delete as appropriate)
33. Do you undertake any activities to prevent entry of FMD into your farm?
    a. Yes (go to question 31)
    b. No (go to question 33)
34. What activities do you normally undertake to prevent foot and mouth disease occurring at your farm (when there is **no outbreak** in the area)?
    a. ______________________________
    b. ______________________________
    c. ______________________________
    d. ____
    e. ______________________________
35. What additional activities do you undertake when **there is an outbreak**?
    a. ____
    b. ___________________________
    c. ___________________________

d. ____
e. ________________________

36. Do you usually vaccinate your animals for FMD?
    a. Yes
    b. No (go to question 34)
    c. Don’t know (go to question 34)
37. How many times in a year do you usually do this? ____________
38. At what age (in months) do you start vaccinating your animals? _________months
39. Who usually vaccinates your animals?
    a. Private veterinarian
    b. Private animal health assistant
    c. Government veterinarian
    d. Government animal health assistant
    e. Other (please state) ____
40. When did you last vaccinate for FMD? (mm/yy)? _______/__________

Milk Production and Delivery

41. How much milk did your farm produce today? _________ litres
42. How much of this did you (or will you) sell? _________ litres
43. Do you sell milk to a cooperative society?
    a. Yes - please provide name ____
    b. No
    c. Don’t know
44. How is the milk transported from the farm?
    a. Farmer delivers direct to dairy
    b. Farmer leaves at a collection point
    c. Milk is collected at farm gate by dairy
    d. Milk is collected at farm gate by hawker
    e. Other (please state) ______________________________
45. How do you preserve the milk until delivery to the dairy?
    a. Refrigeration
    b. Addition of chemicals. If so which ones? ____________________
    c. Immersion in cold water.
    d. No action taken
46. What do you do with the milk during a foot and mouth disease outbreak?
    a. Sell it to the cooperative society
    b. Consume it at home
    c. Sell it to others
    d. Dispose of it
    e. Other (please specify) _____________________________
